# Genome-Wide Comparative Gene Family Classification

**DOI:** 10.1371/journal.pone.0013409

**Published:** 2010-10-15

**Authors:** Christian Frech, Nansheng Chen

**Affiliations:** Department of Molecular Biology and Biochemistry, Simon Fraser University, Burnaby, British Columbia, Canada; American Museum of Natural History, United States of America

## Abstract

Correct classification of genes into gene families is important for understanding gene function and evolution. Although gene families of many species have been resolved both computationally and experimentally with high accuracy, gene family classification in most newly sequenced genomes has not been done with the same high standard. This project has been designed to develop a strategy to effectively and accurately classify gene families across genomes. We first examine and compare the performance of computer programs developed for automated gene family classification. We demonstrate that some programs, including the hierarchical average-linkage clustering algorithm MC-UPGMA and the popular Markov clustering algorithm TRIBE-MCL, can reconstruct manual curation of gene families accurately. However, their performance is highly sensitive to parameter setting, *i.e.* different gene families require different program parameters for correct resolution. To circumvent the problem of parameterization, we have developed a comparative strategy for gene family classification. This strategy takes advantage of existing curated gene families of reference species to find suitable parameters for classifying genes in related genomes. To demonstrate the effectiveness of this novel strategy, we use TRIBE-MCL to classify chemosensory and ABC transporter gene families in *C. elegans* and its four sister species. We conclude that fully automated programs can establish biologically accurate gene families if parameterized accordingly. Comparative gene family classification finds optimal parameters automatically, thus allowing rapid insights into gene families of newly sequenced species.

## Introduction

There are more than 20,000 protein-coding genes in a typical metazoan genome [1]. Although genes differ in sequence, size, and functional domains, they can be grouped into families based on their homology [2]. Genes of the same family usually share similar sequences, functional domains, and even interacting partners. While some gene families are more dynamic in evolution and show species-specific gene members, others are more conserved and found in distantly related species or even across complete kingdoms of life. For example, RFX transcription factors can be found in all mammalian species and each species has exactly seven RFX genes [3]. In contrast, the *srz* chemosensory gene family has different sizes in closely related *Caenorhabditis* species [4].

Gene family classification, *i.e.* the grouping of genes or proteins into families, often yields important insights into gene evolution and gene function [5], [6]. Indeed, arguably the first task biologists do after having cloned a new gene is to examine whether it belongs to a predefined gene family. Ever since the first protein database was established in the 1970s, grant efforts have been made to classify proteins into families for insight into their functional significance. As a result, a large number of gene families, such as the glutamate receptor family [7], the ABC transporter family [8], and many gene families of the G-protein coupled receptor (GPCR) superfamily [9], have been curated primarily experimentally. While the accurate definition of gene families is pivotal for their functional studies, it is very demanding to curate gene families in all sequenced genomes, which often carry similar but different gene families. Thus, automatic classification of gene families is highly desirable.

Necessitated by tens of thousands of genes revealed by genome sequencing projects like the Human Genome Project [10], [11], many sequence-based methods for automated gene family classification have been developed within the last 20 years. These methods can be divided into three major categories. The first category use phylogenetic trees to infer gene families. Phylogenetic tree construction is not easily automated and computationally expensive, which limits its application for genome-wide gene family classification, although recently tree-based methods have been successfully scaled up to multi-genome data sets [12]–[14]. Methods of the second category group genes according to similarities with known sequence signatures like motifs or domains. Sequence signatures are typically derived from manually curated multiple sequence alignments and stored in public databases, such as PROSITE [15], Pfam [16], or SMART [17]. Signature-based methods are routinely used for gene function annotation, but, depending on the method, have different limitations, for example the correct resolution of gene family substructures or the classification of gene families with yet uncharacterized motifs or domains [18]. Methods of the third category, which are in the focus of this project, are based on pairwise comparisons of full-length protein sequences and typically involve the use of clustering techniques [19], [20]. Clustering methods can be applied to classify many sequences in short time, in an automated manner, and most importantly, with reasonable accuracy [21]. Although clustering programs for gene family classification can perform reasonably well in generating gene families, they need to be parameterized for optimal performance. For example, in TRIBE-MCL [21], the inflation value is an important parameter that controls cluster granularity and thus gene family size. However, how to find the right inflation value for the correct resolution of gene families is not intuitively clear. Thus, strategies for using TRIBE-MCL range from simply using program defaults [22] or arbitrary user-defined values [23] to the generation of multiple classifications using different parameter values [24] and the use of parameter values found to be globally optimal with respect to some empirical quality measure [25]. It is expected that different gene families require different cluster granularities for correct resolution. Consequently, neither of the above strategies ensures the quality of classified gene families.

In this paper, we first demonstrate how parameters impact the outcome of clustering-based gene family classification programs, using two sets of highly curated *Caenorhabditis elegans* gene families, the chemosensory and the ABC transporter gene families as example. We find that many programs can indeed achieve very accurate results, but their performance requires careful fine-tuning of parameters to both gene families and data set size. We propose a novel strategy called *comparative gene family classification*, which takes advantage of the existing gene family classification knowledge by automatically calibrating program parameters on reference gene families from well-studied species to classify genes of the same families in related, newly sequenced species. Finally, the effectiveness of this approach is demonstrated by classifying chemosensory and ABC transporter genes across all five sequenced *Caenorhabditis* species and some practical guidelines are given to users interested in comparative gene family classification.

## Results

To appreciate the performance of gene family classification programs, we obtained all published programs for which a stand-alone version was available. Altogether, among 20 published methods, eight were downloaded from websites [21], [26]–[32], while two programs were requested from developers [33], [34]. Of these 10 programs, three were excluded from further analysis because they do not scale well and could not finish the analysis in time (>20 days on one desktop computer) [33], the program is no longer maintained for newer operating systems (Fedora Core 7, kernel 2.6.23.17–88, gcc 4.1.2) [35], or the program requires a license [30]. Thus, the following seven programs were selected for performance assessment (Table 1): TRIBE-MCL [21], gSPC [34], CLUSS [29], FORCE [32], MC-UPGMA [31], HomoClust [28], and BLASTClust [26].

**Table 1:** Identified clustering methods for automated sequence-based gene family classification.

Program	Methodology	Similarity measure	Distant homologs	Multi-domain	Tree cutting	Large-scale	Stand-alone	Evaluated(why not)
**TRIBE-MCL** [21]	Markov CL	BLAST E-value	transitivity	implicit	n/a	yes	yes	yes
**gSPC** [34]	superparamagnetic CL	BLAST E-value	n/d	n/d	n/a	yes	yes	yes
**BLASTClust** [26]	single-linkage CL	BLAST score	transitivity	no	n/a	yes	yes	yes
**HomoClust** [28]	single-linkage CL	BLAST E-value	transitivity	implicit	no	yes	yes	yes
**FORCE** [32]	graph-based CL	BLAST E-value	n/d	implicit	n/a	no	yes	yes
**CLUSS** [29]	average-linkage CL	shared subseq.	n/d	implicit	yes	no	yes	yes
**MC-UPGMA** [31]	average-linkage CL	BLAST E-value	n/d	implicit	no	yes	yes	yes
**SYSTERS** [52], [53]	single-linkage CL	BLAST E-value	transitivity	no	yes	yes	no	not available
**ProtoNet** [54], [55]	average-linkage CL	BLAST E-value	transitivity	implicit	yes	yes	no	not available
**GeneRAGE** [33]	single-linkage CL	SW Z-score	transitivity	explicit	n/a	no	yes	long runtime
**CluSTr** [56]	single-linkage CL	SW Z-score	transitivity	no	no	yes	no	not available
**Picasso** [57]	profile alignment	BLAST E-value	trans., profiles	explicit	n/a	no	no	not available
**ProClust** [27], [35]	graph-based CL	SW E-value	trans., HMMs	implicit	n/a	no	yes	compile errors
**Ncut** [58]	graph-based CL	BLAST E-value	transitivity	explicit	n/a	n/d	no	not available
**Paccanaro ** ***et al.*** [59]	spectral CL	LR probability	n/d	n/d	n/a	yes	no	not available
**Harlow ** ***et al.*** [60]	MCL+single-linkage CL	BLAST bitscore	transitivity	implicit	no	yes	no	not available
**JACOP** [61]	average-linkage CL	shared subseq.	no	implicit	no	no	no	not available
**CLUGEN** [62]	average-linkage CL	NN score	transitivity	implicit	no	n/d	no	not available
**BAG** [30]	graph-based CL	FASTA E-value	transitivity	implicit	n/a	n/d	yes	license requ.
**SEQOPTICS** [47]	density-based CL	SW score	no	implicit	n/a	no	no	not available

Evaluated methods are listed first and the two resulting sub-lists are then sorted chronologically by publication date (older methods first). Methods are categorized according to classification methodology, protein sequence similarity measure, and if and how key challenges of gene family classification are addressed. *Distant homologs* indicates if and how detection of remote homologs is addressed. *Multi-domain* indicates if and how the problem of multi-domain proteins and promiscuous domains is addressed. *Tree cutting* applies to hierarchical clustering techniques only and refers to the functionality of automatically cutting the hierarchical tree of nested clusters into a final, distinct set of putative protein families. *Large-scale* indicates if larger proteome-scale data sets (>20,000 proteins) can be processed on a desktop computer in reasonable time (hours but not days). *Standalone* indicates whether the program is available as stand-alone application and can be installed and run locally. *Evaluated* indicates if the method was amenable for performance evaluation in this paper and why not if otherwise. Abbreviations: n/a: not applicable; n/d: not determined; SL: single-linkage; CL: clustering; LR: logistic regression; NN: neural network; SW: Smith-Waterman; MCL: Markov clustering; HMM: hidden markov model.

Gene family classification performance was tested using two *C. elegans* gene families that have been extensively curated. The first data set comprises the 22 *C. elegans* chemosensory gene families (Table S1). Chemosensory genes play an important role in the chemosensation of soil nematodes and constitute the largest known gene superfamily in *C. elegans* with about 1,300 putatively functional genes [36]. Chemosensory genes belong to the broader class of G protein-coupled receptors (GPCRs) and share the universal characteristic of a seven transmembrane domain (7-TM) structure [37]. Chemosensory gene families in *C. elegans* have undergone extensive bioinformatics analysis. Indeed, all chemosensory gene families in *C. elegans* have been extensively and manually curated by many groups in the last decade [4], [23], [36]–[43]. Twenty-two different *C. elegans* chemosensory gene families have been curated, ranging from the large *srh* and *str* families that comprise about 200 putative functional genes [39], [40] to the single-gene family *srn* [36]. Comparative analysis of chemosensory genes in *C. elegans* and *C. briggsae* suggests that chemosensory genes are very dynamic in evolution and many genes are species-specific. The second data set consists of the eight *C. elegans* ABC transporter gene families (Table S2). In contrast to the actively evolving chemosensory gene families, ABC transporter gene families are highly conserved and are found in many species. These genes are mostly involved in substrate transport across membranes. ABC transporters are classified into eight families (A to H) based on number and order of transmembrane and ATP-binding domains [44]. In *C. elegans*, 60 ABC transporter genes have been identified [44], [45].

### Gene family classification programs successfully reconstruct curated classifications

We tested these seven programs for their ability to reconstruct the classification of both chemosensory genes and ABC transporter genes. Each program was run with the complete *C. elegans* proteome (WS180) as input, which contains 20,140 protein products. Only the longest isoform for each gene was classified, since different isoforms belong to the same gene family. To allow for a fair performance assessment, all programs except CLUSS were provided with an identical pair-wise protein sequence similarity matrix for clustering. We computed pair-wise similarities in an all-vs-all BLAST search with an E-value cut-off value of 1e-10. CLUSS implements an alignment-independent similarity measure [29] and therefore was run directly with the *C. elegans* protein sequences as input. To achieve the best performance for both chemosensory gene families and ABC transporter gene families for each program, we systematically tested a range of different parameters (see Materials and Methods). The best result was considered as those that gave the best overlap with all gene families in a reference data set in terms of the highest weighted average Jaccard index [46] (Figure S1). The Jaccard index accounts for both sensitivity and specificity of a classification result and was used previously for performance evaluation [31], [47], [48].

**Figure 1:**
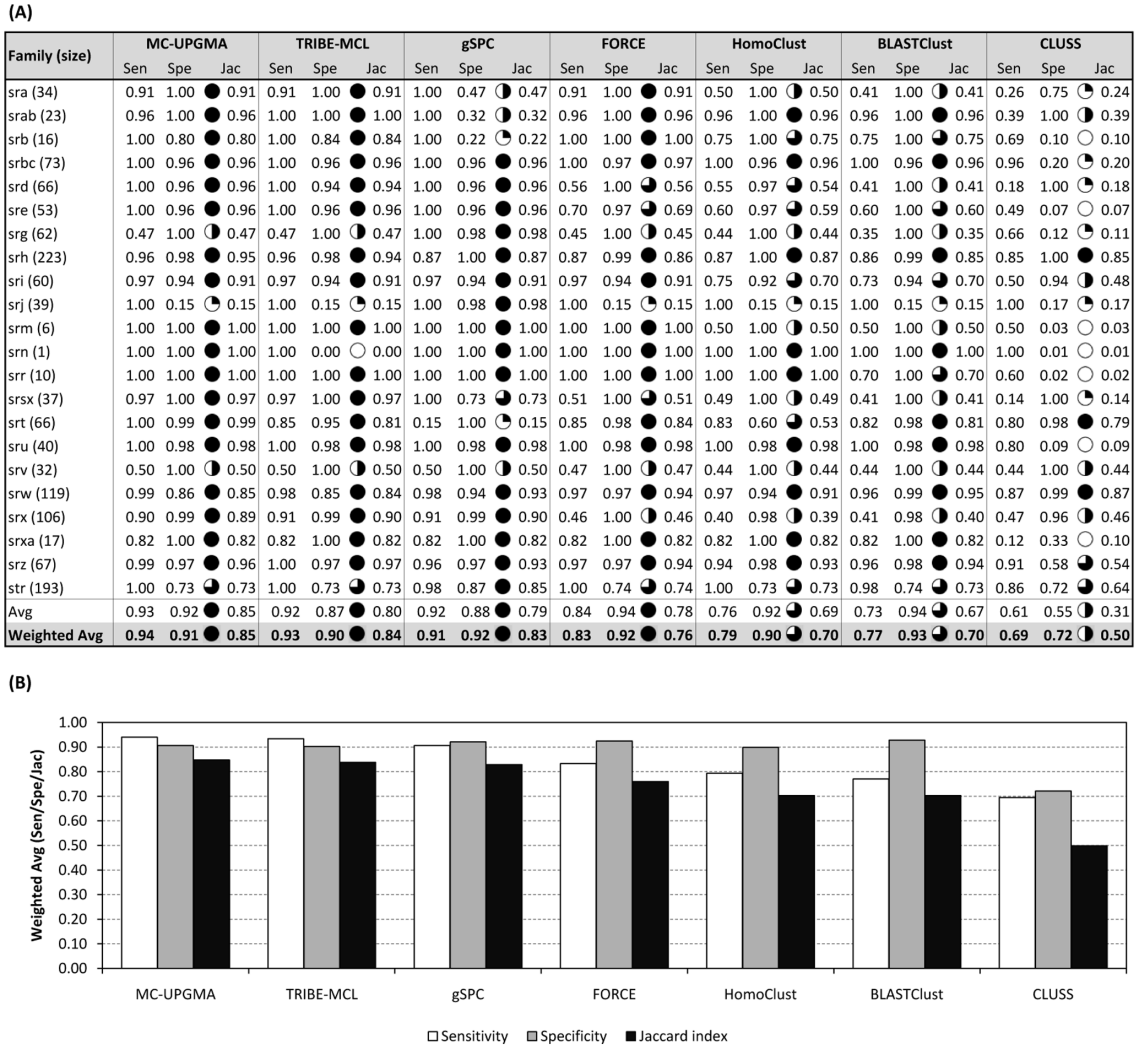
Classification performance for each *C. elegans* chemosensory gene family (A) and weighted average over all 22 chemosensory gene families (B). MC-UPGMA shows best performance on average, closely followed by TRIBE-MCL and gSPC. For each method, the complete *C. elegans* proteome (WS180, 20,140 proteins) was clustered with different parameters, and the result with the highest weighted average Jaccard index over all 22 chemosensory gene families is shown here. Filled circles correspond to adjacent Jaccard indices: full  =  Jac >0.75; three-quarter  =  Jac >0.5; half  =  Jac >0.25; quarter  =  Jac >0.1; empty  =  Jac ≤0.1. *Avg* refers to the unweighted average (arithmetic mean) of family-specific performance values, and *Weighted Avg* refers to averages weighted by family size. Abbreviations: Sensitivity (Sen); Specificity (Spe); Jaccard index (Jac).

**Figure 2:**
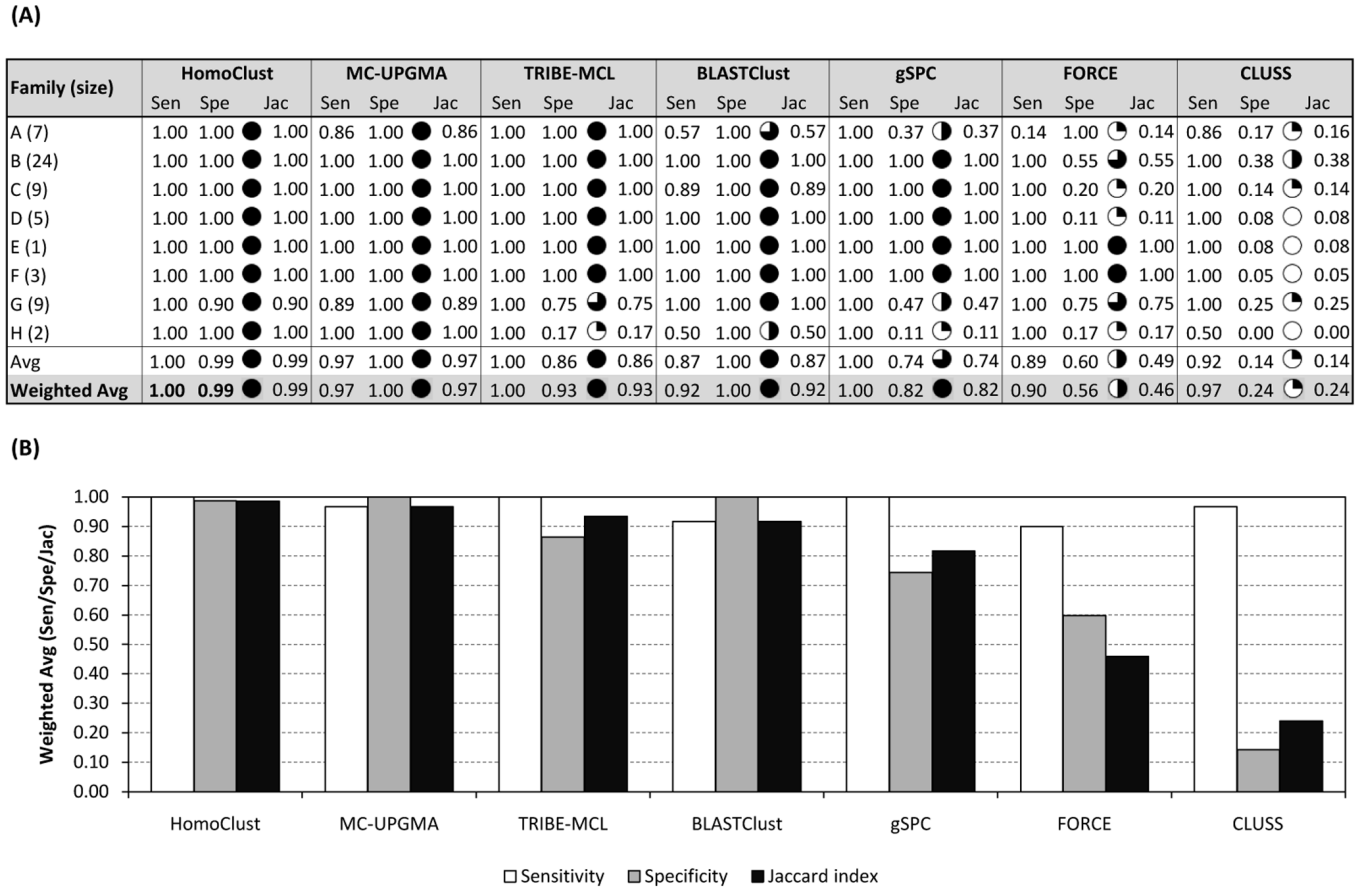
Classification performance for each *C. elegans* ABC transporter gene family (A) and weighted-average over all eight ABC transporter gene families (B). HomoClust performs best in terms of weighted average Jaccard index, closely followed by MC-UPGMA and TRIBE-MCL. Classification procedure was the same as in Figure 1, except that program parameters were optimized for ABC transporter gene families. Legend and abbreviations as in Figure 1.

For the chemosensory genes, three programs, MC-UPGMA, TRIBE-MCL, and gSPC, reproduce the manual classification with high quality and clearly outperform other programs (Figure 1). In particular, MC-UPGMA performs best on the chemosensory gene data set (weighted average Jaccard index  = 0.85), followed by TRIBE-MCL (0.84), gSPC (0.83), FORCE (0.76), HomoClust and BLASTClust (both 0.70). CLUSS performs poorly on chemosensory genes, with a weighted average Jaccard index of 0.50. For the ABC transporter genes, four programs, HomoClust, MC-UPGMA, TRIBE-MCL, and BLASTClust, clearly outperform others (Figure 2). The best result is achieved by HomoClust, which groups ABC transporter genes almost perfectly (weighted average Jaccard index 0.99). HomoClust is followed by MC-UPGMA (0.97), TRIBE-MCL (0.93), BLASTClust (0.92), gSPC (0.82), FORCE (0.46), and CLUSS (0.24). Thus, we conclude from this analysis that fully automated computer programs can fairly faithfully reconstruct most of the highly curated chemosensory and ABC transporter gene families.

Considering classification results on chemosensory genes (Figure 1) and on ABC transporter genes (Figure 2) together, we can see that certain programs tend to outperform others. In particular, MC-UPGMA and TRIBE-MCL give good results on both reference sets. For chemosensory genes, MC-UPGMA performs significantly better than FORCE (p = 0.033, one-sided paired t-test), HomoClust (p = 0.001), BLASTClust (p = 0.001), and CLUSS (p = 2.1e-7). TRIBE-MCL significantly outperforms BLASTClust (p = 0.044) and CLUSS (p = 1.5e-6). For ABC transporter genes, both MC-UPGMA and TRIBE-MCL significantly outperform FORCE (p = 0.0048 and 0.02, respectively) and CLUSS (p = 5.6e-7 and 7.7e-5, respectively). TRIBE-MCL and MC-UPGMA perform similarly well on both data sets (chemosensory genes: p = 0.27; ABC transporter genes: p = 0.184). 13 chemosensory gene families are grouped equally well by both methods, six (including the single-gene family *srn*) are grouped slightly better by MC-UPGMA, and three are grouped slightly better by TRIBE-MCL (Figure 1). Four chemosensory gene families (*srg*, *srj*, *srv,* and *str*) remain poorly grouped by both MC-UPGMA and TRIBE-MCL, from which one gene family (*srv*) represents a challenge for all evaluated methods (see Text S1 as well as Figure S2 and Figure S3). TRIBE-MCL tends to produce larger clusters that contain more than one gene family, as is exemplified by the chemosensory single-gene family *srn,* which is grouped together with the large *srh* family. Similarly, TRIBE-MCL grouped the two ABC transporter gene families G and H together.

Overall, both MC-UPGMA and TRIBE-MCL can fairly faithfully reconstruct most chemosensory and ABC transporter gene families. In addition, these two methods perform better than others on two very different data sets. In the following experiments, we will use MC-UPGMA and TRIBE-MCL to illustrate the idea that parameter tuning of gene family classification programs is essential for their performance.

### Program parameters need tuning for different gene families

Although MC-UPGMA and TRIBE-MCL nicely reproduce manually curated gene families from both data sets, quite different parameters were required to achieve optimal performance. For chemosensory genes, MC-UPGMA performs best if the cluster hierarchy (the tree) is cut at E-value 9.6. TRIBE-MCL achieves best results on the same data set with inflation value 1.2. In contrast, for ABC transporter gene families, the optimal tree cut-off value for MC-UPGMA is at E-value 1e-14, and the optimal inflation value for TRIBE-MCL is 2.6.

**Figure 3:**
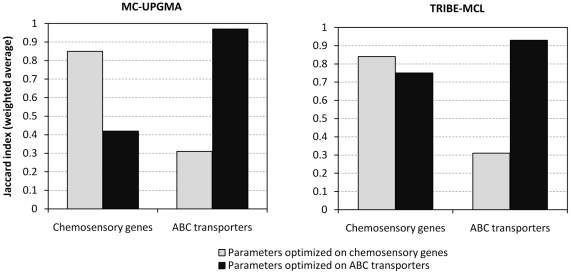
Clustering parameters optimal for chemosensory genes give poor performance on ABC transporters and vice versa. The left panel shows classification performance achieved by MC-UPGMA on both chemosensory genes and ABC transporters if parameters optimal for chemosensory genes (grey bars; tree-cutoff = 9.6) and ABC transporters (black bars; tree-cutoff = 1e-14) are used. The right panel shows the same for TRIBE-MCL, with grey bars and black bars corresponding to inflation values of 1.2 and 2.6, respectively. For both programs performance drops significantly if parameters are optimized on the respective other data set.

The performance of both programs deteriorates if we use parameters tuned for one data set for classifying the respective other (Figure 3). The weighted average Jaccard index for MC-UPGMA drops from 0.85 to 0.42 on chemosensory genes and from 0.97 to 0.31 on ABC transporter genes. Similarly, TRIBE-MCL drops from 0.84 to 0.75 on chemosensory genes and from 0.93 to 0.31 on ABC transporter genes. Clearly, there is no single parameter set that performs well on both data sets, which suggests that the performance of both programs depends on the tuning of parameters for different types of gene families.

### Performance of gene family classification programs also depends on data set size

The performance of gene family classification programs depends not only on gene families, but also on the size of the data set. This phenomenon becomes evident when we run MC-UPGMA and TRIBE-MCL on a larger data set consisting of proteomes of five nematode species, including *C. elegans, C. briggsae*, *C. remanei*, *C. japonica*, and *C. brenneri* (130,208 proteins in total).

**Figure 4:**
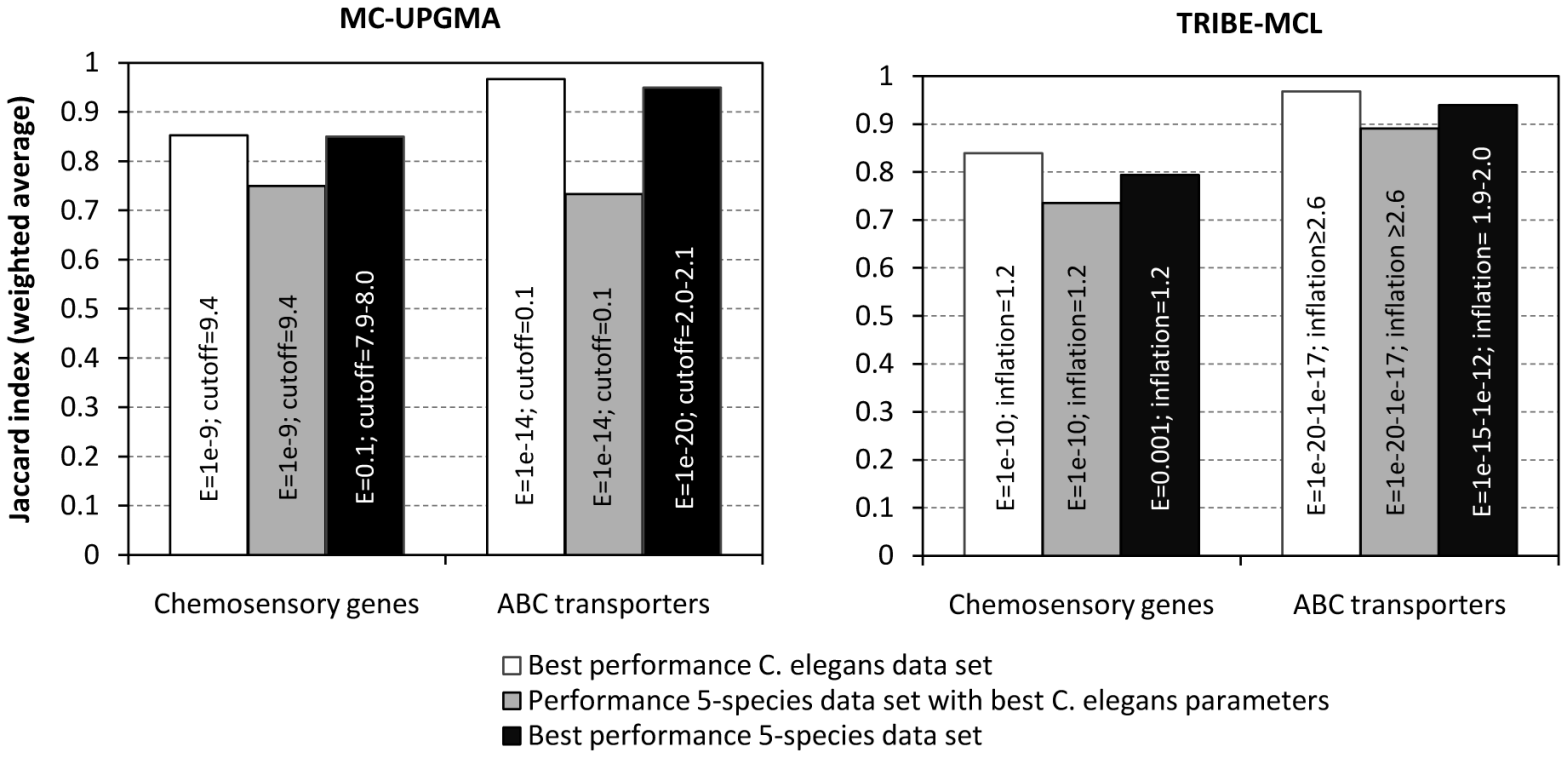
Classification parameters of MC-UPGMA and TRIBE-MCL need to be adjusted for data set size. Left and right panel show the best classification performance achieved by MC-UPGMA and TRIBE-MCL, respectively, if clustering is performed on the *C. elegans* proteome in isolation (white bars), on the *Caenorhabditis* five-species data set with unchanged parameters (grey bars), and on the *Caenorhabditis* five-species data set with readjusted parameters (black bars). Optimal clustering parameters shown within bars. A range of parameter values indicates that multiple parameter settings achieved equal top performance. Note that for this comparison we treated the BLAST E-value threshold used for filtering protein sequence similarities prior to classification as an additional parameter that was allowed to vary (between 1e-50 and 0.1).

Using MC-UPGMA with parameters optimal for classifying chemosensory genes in the *C. elegans* data set only, we observe a drop in performance when classifying the same genes in the context of the larger, five-species data set (Figure 4). The weighted average Jaccard index drops from 0.85 to 0.75 (p = 0.016, one-sided paired t-test). For example, the chemosensory gene family *sre* is now split into two families, one with 15 *sre* genes and the other with 38 genes. Similarly, gene family *srsx* classified almost perfectly on the *C. elegans*-only data set before (Jaccard index 0.97) is now roughly split into half, with 17 *srsx* genes in one family and 19 in the other. The performance decrease of MC-UPGMA is even more pronounced on ABC transporters, where the average weighted Jaccard index drops from 0.97 to 0.73 (p = 0.009, one-sided paired t-test) on the five-species data set. A similar problem is observed for TRIBE-MCL, which drops in performance from 0.84 to 0.74 on chemosensory genes (p = 0.146, one-sided paired t-test), and from 0.97 to 0.89 on ABC transporter genes (p = 0.052).

However, the lower performance of both programs can be improved by further parameter tuning for both types of genes. After readjusting program parameters to the larger data set, the performance of both programs, MC-UPGMA and TRIBE-MCL, improves to similar levels as seen before on the *C. elegans* proteome (Figure 4).

### Comparative gene family classification—proposing a new gene classification strategy

We have demonstrated that fully automated programs for gene family classification can reproduce curated gene families. However, program parameters are critical for classification performance and need tuning for optimal results. Since there is no “one-parameter-fits-all” strategy in gene family classification, and optimal parameters are not known *a priori*, how should we use gene family classification programs?

Here, we propose a comparative gene family classification strategy that takes advantage of the availability of curated, high-quality gene families in well-studied species to classify proteins of these families in related, less-studied species. For example, we can classify gene families in the *Caenorhabditis* species by taking advantage of the curated gene families in *C. elegans*. In the first step of this strategy, proteins of one or more species of interest are pooled into one large data set. This data set is then classified with parameters chosen such that classification performance is maximized on the curated, known gene families. Proteins of different species found in identical clusters are then classified as belonging to the same families.

To demonstrate the usability of this approach, we used TRIBE-MCL to classify both chemosensory genes and ABC transporter genes in a combined data set containing proteins from all five sequenced *Caenorhabditis* species, which are available at WormBase (http://www.wormbase.org) release WS204 (130,208 proteins in total). We identified parameters that generate the best classification of *C. elegans* chemosensory gene families, which we found to be E-value threshold  = 0.001 and inflation value  = 1.2. Notably, these parameters are different from those that generated the best classification of *C. elegans* chemosensory genes for the *C. elegans* proteome alone (Figure 4). Using TRIBE-MCL and these parameters, we classified chemosensory genes in all five *Caenorhabditis* species (Table 2 and Table S3).

**Table 2:** Increased chemosensory gene content in *C. elegans* and *C. remanei* and greatly reduced gene content in *C. japonica*.

Gene family (size)	Cele	Cbri	Cbre	Crem	Cjap	TP	FP	FN	Sen	Spe	Jac
sra (34)	31	21	22	26	13	31	0	3	0.91	1.00	0.91
srab (23)	27	18	14	28	15	23	4	0	1.00	0.85	0.85
srb (16)	16	17	15	24	11	16	0	0	1.00	1.00	1.00
srbc (73)	88	42	47	40	11	73	15	0	1.00	0.83	0.83
srd (66)	69	47	57	64	66	66	3	0	1.00	0.96	0.96
sre (53)	60	47	60	64	31	53	7	0	1.00	0.88	0.88
srg (62)	57	65	77	94	39	28	29	34	0.45	0.49	0.31
srh (223)	221	145	166	226	68	215	6	8	0.96	0.97	0.94
sri (60)	63	47	37	82	28	58	5	2	0.97	0.92	0.89
srj/str (232)	268	239	247	353	98	232	36	0	1.00	0.87	0.87
srm (6)	16	17	20	32	12	6	10	0	1.00	0.38	0.38
srn (1)	1	1	0	1	1	1	0	0	1.00	1.00	1.00
srr (10)	10	8	9	11	5	10	0	0	1.00	1.00	1.00
srsx (37)	48	45	41	43	39	36	12	1	0.97	0.75	0.73
srt (66)	58	47	68	75	17	57	1	9	0.86	0.98	0.85
sru (40)	41	39	58	103	14	40	1	0	1.00	0.98	0.98
srv (32)	16	17	17	32	7	16	0	16	0.50	1.00	0.50
srw (119)	153	121	128	163	42	117	36	2	0.98	0.76	0.75
srx (106)	99	89	57	111	39	96	3	10	0.91	0.97	0.88
srxa (17)	3	3	1	2	2	3	0	14	0.18	1.00	0.18
srz (67)	69	39	70	110	0	67	2	0	1.00	0.97	0.97
**All (1,343)**	**1,414**	**1,114**	**1,211**	**1,684**	**558**	**1,213**	**170**	**99**	**0.93**	**0.89**	**0.84**

Known *C. elegans* chemosensory gene families (WS180, leftmost column) are shown next to cluster sizes as determined by clustering a pooled data set consisting of five *Caenorhabditis* proteomes with TRIBE-MCL. The combined data set was clustered with different parameters, and shown is the result with the best overlap (i.e. highest unweighted Jaccard index) with known *C. elegans* chemosensory gene families (E-value threshold = 0.001; inflation value = 1.2). The remaining columns quantify the quality of overlap with *C. elegans* families: true-positives (TP), false-positives (FP), and false-negatives (FN), sensitivity (Sen), specificity (Spe), and Jaccard index (Jac). Values in the last row represent sums, except for Sen/Spe/Jac values where they correspond to average values weighted by family size. Note that gene families *srj* and *str* were clustered together by TRIBE-MCL and gene numbers of both families are combined for cross-species comparison. Abbreviations: Cele: *C. elegans*; Cbri: *C. briggsae*; Cbre: *C. brenneri*; Crem: *C. remanei*; Cjap: *C. japonica*.

As expected, large numbers of chemosensory genes are found in all five sequenced *Caenorhabditis* species. As previously reported, there are more chemosensory genes in *C. elegans* (1,414 genes) compared to *C. briggsae* (1,114 genes) [23], [38]. In addition, our comparative gene family identification strategy suggests that *C. remanei* (1,684 genes) has more chemosensory genes than any other *Caenorhabditis* species whose genomes has been sequenced, with some pronounced family size increases relative to *C. elegans,* such as *srb* (+50%) and *sru* (+150%). *C. brenneri* (1,211 genes) has a similar number of chemosensory genes as *C. briggsae*, while *C. japonica* (558 genes) has the least chemosensory genes compared to the other four *Caenorhabditis* species. Some of the chemosensory gene family sizes in *C. remanei* may be overestimated since it has been demonstrated that genome sequences used for sequencing were extracted from heterozygotes [49]. The sequenced *C. brenneri* genome might contain heterozygosity as well. The low number of chemosensory genes in the *C. japonica* genome partly reflects that the genome sequence contains gaps, which cover ∼20% of the genome. These missing regions may be enriched with chemosensory genes. An interesting difference for *C. japonica* is observed for the previously mentioned *srz* gene family, which it seems to lack entirely. Another potentially confounding factor that should be kept in mind is that the gene models predicted for the three *Caenorhabditis* species *C. remanei*, *C. brenneri*, and *C. japonica* are preliminary and have not been examined closely. Therefore, many gene models may not be accurate and many others might still be missing.

Using the same strategy, we classified ABC transporter genes in all five *Caenorhabditis* species (Table 3 and Table S4). Contrary to chemosensory genes, *C. elegans* shows the lowest number of ABC transporter genes overall (61 genes), which is slightly less than *C. briggsae* (73 genes) and *C. remanei* (73 genes). *C. brenneri* has the highest number of genes (102 genes; +67% relative to *C. elegans*). *C. japonica* shows also an increased gene content for most ABC transporter gene families relative to *C. elegans* (87 genes in total; +43%).

**Table 3:** Increased numbers of ABC transporter genes in *Caenorhabditis* species compared to *C. elegans*.

Gene family (size)	Cele	Cbri	Cbre	Crem	Cjap	TP	FP	FN	Sen	Spe	Jac
A (7)	6	6	12	6	13	6	0	1	0.86	1.00	0.86
B (24)	24	32	37	30	34	24	0	0	1.00	1.00	1.00
C (9)	10	14	22	13	9	9	1	0	1.00	0.90	0.90
D (5)	5	4	7	5	9	5	0	0	1.00	1.00	1.00
E (1)	1	1	1	1	1	1	0	0	1.00	1.00	1.00
F (3)	3	4	5	6	6	3	0	0	1.00	1.00	1.00
G (9)	11	11	15	11	12	9	2	0	1.00	0.82	0.82
H (2)	1	1	3	1	3	1	0	1	0.50	1.00	0.50
**All (60)**	**61**	**73**	**102**	**73**	**87**	**58**	**3**	**2**	**0.97**	**0.96**	**0.92**

Known *C. elegans* ABC transporter gene families [45] are shown next to cluster sizes as determined by clustering a pooled data set consisting of five *Caenorhabditis* proteomes with TRIBE-MCL. The combined data set was clustered with different parameters, and shown is the result with the best overlap (i.e. highest unweighted Jaccard index) with known *C. elegans* ABC transporter gene families (E-value threshold = 1e-20; inflation value = 1.9). *Overlap C. elegans* quantifies the quality of overlap with known *C. elegans* families: true-positives (TP), false-positives (FP), false-negatives (FN), sensitivity (Sens), specificity (Spec), and Jaccard index (Jacc). Abbreviations: Cele: *C. elegans*; Cbri: *C. briggsae*; Cbre: *C. brenneri*; Crem: *C. remanei*; Cjap: *C. japonica*.

### Comparative gene family classification gives novel insights into well-studied gene families

Here we demonstrate that automatically generated classification results provide an excellent starting point for further in-depth analysis of genes and gene families. The expansion and contraction of some gene families may be genuine, while others may reflect defective curation, thus incorrect classification. For example, the ABC transporter family C, which was analyzed extensively by Zhao and colleagues among the three nematode species *C. elegans*, *C. briggsae*, and *C. remanei* [45], showed some conspicuous differences between our classification and previous analysis. Zhao *et al.* found nine genes in each of these species with clear one-to-one orthology relationships. In contrast, our analysis suggested a larger number of putative ABC transporter C genes: 10 in *C. elegans*, 14 in *C. briggsae*, and 13 in *C. remanei* (Table 3). These differences in gene numbers motivated us to investigate the automatic classification result of ABC transporter family C in more detail.

**Figure 5:**
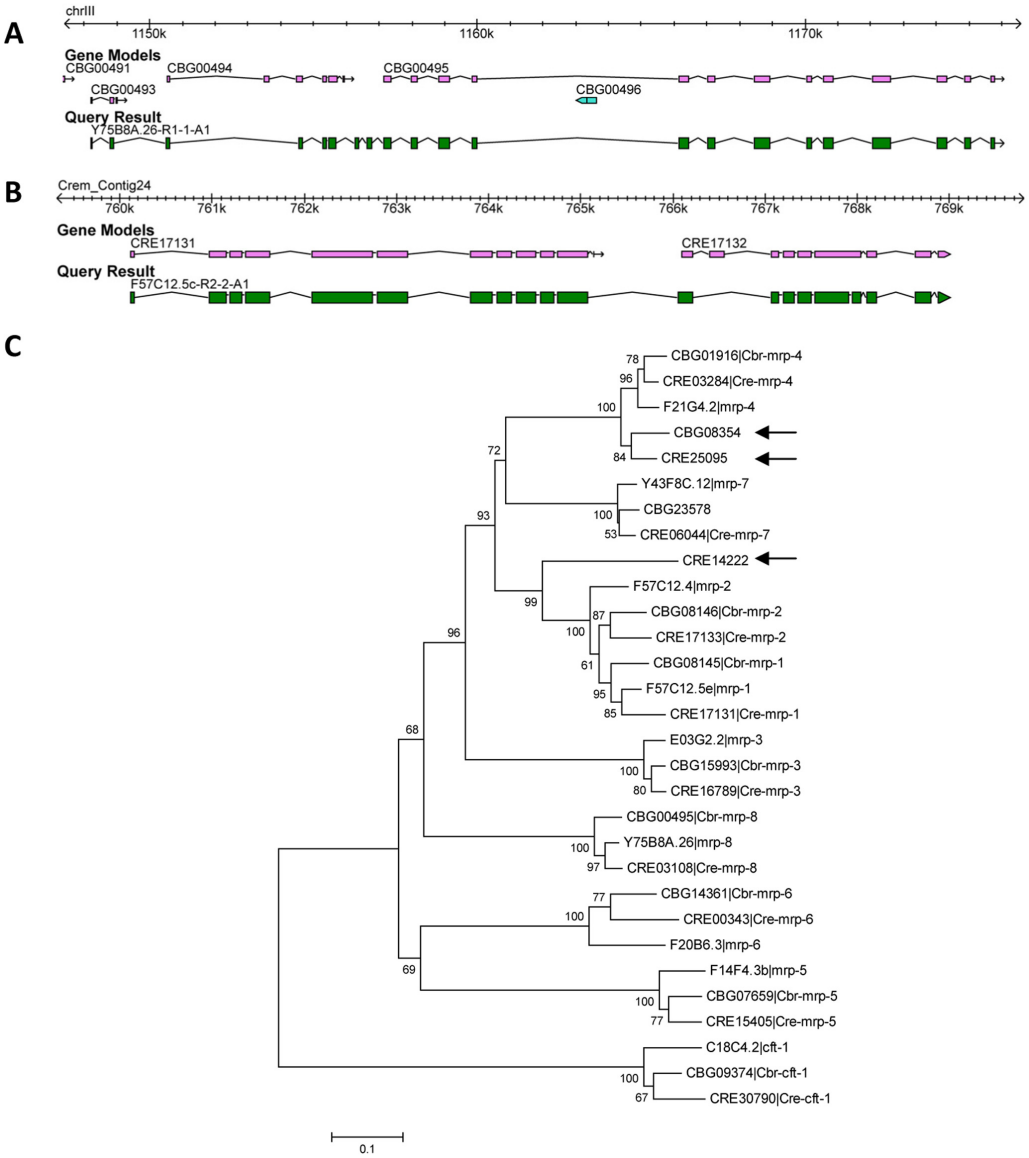
Improved ABC transporter gene models in *C. briggsae* (A) and *C. remanei* (B) and phylogenetic tree positioning the three newly identified genes within ABC transporter gene family C (C). Panel A shows the current gene model of *C. briggsae* gene CBG000495 (*Cbr-mrp-8*) as well as the improved gene model obtained by running genBlastG with default parameters using *C. elegans* ortholog Y75B8A.26 as query (http://genome.sfu.ca/genblast/). Panel B shows both current and improved gene model for the *C. remanei* gene CRE17131 (*Cre-mrp-1*) using *C. elegans* ortholog F57C12.5c (longest confirmed isoforms) as query. The phylogenetic tree shows the evolutionary relationship of the three new ABC transporter genes CBG08354, CRE25095, and CRE14222 (indicated by arrows) with known *C. elegans*, *C. briggsae*, and *C. remanei* ABC transporters of family C. Tree is drawn to scale (number of substitutions per site). Numbers at branch points represent bootstrap values from 1,000 iterations.

Analysis of these differences revealed novel *bona fide* ABC transporters (Figure 5), as well as defective gene models in current gene annotations of *Caenorhabditis* genomes. Three genes classified by TRIBE-MCL as ABC transporters have all necessary domains (CBG08354 in *C. briggsae* and CRE14222 and CRE25095 in *C. remanei*). These three genes were missed in previous analyses likely because of improved *C. briggsae* and *C. remanei* contig assembly after the work of Zhao *et al*. was finished (personal communication). In a phylogenetic analysis, the three new genes group nicely within known ABC transporters of family C (Figure 5C). Comparing three *C. briggsae* gene models CBG00493, CBG00494, and CBG00495, which were identified by TRIBE-MCL as ABC genes, with known ABC genes suggests that these three models represent fragments of one single ABC gene. Thus these gene models are defective. By running genBlastG, a newly developed comparative gene predictor in our lab (She, Chu, Wang, and Chen, unpublished), we predicted a new gene model that merges these three genes nicely into one gene model (Figure 5A). Similarly, *C. remanei* gene model CRE17132 (503 aa) is a predicted 3′ gene fragment of known ABC transporter gene *Cre-mrp-1* (CRE17131; 893 aa) (Figure 5B).

The remaining difference of 4 genes in the ABC transporter C family between our automatically generated results and those from Zhao *et al.* can be explained by potential errors in the current gene annotation of *C. briggsae*. We encountered a trio of orthologous genes present in family C that are clearly not ABC transporters (C06G4.4, CBG22944, and CRE06748). Inspection of the BLAST similarity matrix revealed that none of these three genes exhibits sequence similarity with known ABC transporters in family C, which raised the question why TRIBE-MCL assigned these three genes to this family. Closer investigation revealed that one *C. briggsae* gene (CBG24505) in the TRIBE-MCL cluster exhibits local sequence similarity both to ABC transporter genes (N-terminal) and to this orthologous trio (C-terminal). Thus CBG24505 functions as linker gene that prompts TRIBE-MCL to pull two otherwise unrelated gene families together. The existence of two alternative, shorter gene models at the locus of CBG24505 suggests that the current gene model of CBG24505 is in fact a fused gene that should be split. One shorter version of CBG24505 encodes for an ABC transporter transmembrane region that has high sequence similarity to *C. elegans* gene *mrp-7* (data not shown). Interestingly, the current (non-adjacent) *C. briggsae* ortholog of *mrp-7,* CBG23578, lacks this 5′ transmembrane region, which suggests either a chromosomal rearrangement or an assembly error in the *C. briggsae* genome that split the *mrp-7* ortholog CBG23578 into separate genes.

Taken together, our comparative gene family classification approach could successfully reproduce previously established gene numbers of the ABC transporter C family in *C. elegans*, *C. briggsae*, and *C. remanei*. In addition, it led to the discovery of both defective gene models and previously missed ABC transporter genes. This confirms the feasibility of this approach and highlights its potential for giving novel and rapid insights into gene families across multiple species.

### Guidelines for tuning parameters for comparative gene family classification

Some practical guidelines can be followed for tuning parameters. Comparative gene family classification is useful if gene families are to be compared across species and if reference classifications exist for at least one of the species. TRIBE-MCL and MC-UPGMA are two programs that are both efficient and accurate for gene family classification in eukaryotes. The Jaccard index is an easy to compute and yet effective measure of cluster quality that can be used to find optimal program parameters. The two main parameters to tune are the inflation value in case of TRIBE-MCL and the E-value tree-cutoff in case of MC-UPGMA. After trying different parameter values the one that yields the highest Jaccard index should be used for classification. For larger datasets, the computation of the pair-wise similarity matrix with BLAST might be the computationally most expensive step of the analysis, but can be easily parallelized if required.

## Discussion

Based on our assessment of publicly available gene family classification programs, we conclude that many are performing well. MC-UPGMA and TRIBE-MCL performed exceptionally well in our comparison and nicely reconstructed most manual classifications of both chemosensory and ABC transporter genes. We attribute the overall outperformance of MC-UPGMA and TRIBE-MCL to beneficial intrinsic properties of the two clustering algorithms. MC-UPGMA utilizes average-linkage clustering, which determines the relatedness of two clusters by taking the mean similarity across all data points in those clusters. Average-linkage clustering is known to be more robust against outliers [31]. We could clearly observe this phenomenon, where for example in comparison to MC-UPGMA the single-linkage clustering algorithm BLASTClust produced clusters of much lower specificity (*i.e.* too large clusters) at similar levels of sensitivity (data not shown). Similarly, the iterative graph-based clustering procedure implemented in TRIBE-MCL is robust against merging clusters that share only few edges, which allows the robust identification of true gene families even in the presence of lower-quality BLAST hits or promiscuous domains [21]. Additional information about clustering methodology and performance of all seven tested programs is provided in Text S1.

Nevertheless, the good performance of gene family classification programs depended on the tuning of program parameters. For example, the overall good performance of both TRIBE-MCL and MC-UPGMA required parameter adjustment for both types of gene families and proteome size. Parameters optimal for classifying one type of gene family yielded poor performance when used for classifying the other (Figure 3). The optimal inflation value for TRIBE-MCL was 2.6 for the ABC transporter genes instead of 1.2 for the chemosensory genes, because lower inflation values cause TRIBE-MCL to incorrectly cluster the more conserved ABC transporter genes into fewer and bigger clusters. An equally dramatic difference was seen for MC-UPGMA in terms of the optimal E-value tree-cutoff for classifying chemosensory genes (E = 9.6) and ABC transporter genes (E = 1e-14), which again reflects the higher sequence divergence among chemosensory genes than among ABC transporter genes. The influence of data set size on classification performance was less pronounced but still substantial (Figure 4). Keeping the E-value for constructing the BLAST similarity matrix constant, we observed the tendency that the larger data set required less stringent clustering parameters for the correct resolution of our gene families; that is, higher inflation values in case of TRIBE-MCL and lower E-value tree-cutoffs in case of MC-UPGMA (data not shown). One possible explanation is that the inclusion of divergent family members from other species leads to more sparsely connected clusters that need less stringent parameters for correct resolution. Thus, taken together, despite the encouraging finding that fully automated programs can reconstruct manually established gene families with good quality in principle, the question remained how these programs should be parameterized in practice.

We proposed a novel, comparative approach to automated gene family classification that takes advantage of already established gene family classifications in one species (reference gene families) to classify genes of the same families in other, related species. Many model organisms are well studied today, and many gene families of these species have been curated in great detail. This existing knowledge of gene families can be readily leveraged for what we call ‘comparative gene family classification’: complete proteomes of well-studied and related species are pooled together and classification parameters are chosen such that classification performance is maximized on the reference gene families. Genes found within identical clusters are then considered as members of the same family. This strategy can be completely automated and thus provides a convenient shortcut to gene family classification within the fast growing body of fully sequenced species, at least at a first approximation. It is worth mentioning that the reference gene families used for parameter calibration must not necessarily be complete, in which case our classification strategy should reveal missing family members within the reference gene family itself. Also, in principal, there is no minimum phylogenetic distance of compared species required for this approach. Comparative gene family classification will consider genes from other species as family members as long as those genes are as closely related to the reference gene family as existing family members are related among each other.

We applied this comparative gene family classification approach to chemosensory genes and ABC transporter genes across all five sequenced *Caenorhabditis* species (Table 2 and 3). We observed a less dramatic increase of chemosensory gene content in *C. elegans* relative to *C. briggsae* than reported previously (+30% instead of +70% in [23] and +40% in [37]), probably due to a constantly improving annotation of the *C. briggsae* genome. Results from other studies that carefully worked up the differential chemosensory gene content between *C. elegans* and *C. briggsae* in selected chemosensory gene families are in good agreement with our results, suggesting that our strategy works well. The reported numbers for the *sra* and *srab* gene families in [38] match almost perfectly with our results, and we observe a similar increase in *C. elegans srz* gene numbers relative to *C. briggsae* (+80%) as reported in [4] (+106%). However, the increased number of chemosensory genes in *C. remanei* relative to *C. elegans* is inconsistent with previous findings [37] and demands explanation. First, we noticed that *C. remanei* has in general an elevated number of predicted genes in its genome (31,518; WS204) relative to *C. elegans* (20,140; WS180, only longest isoforms) and *C. briggsae* (21,978), which is probably attributable to many partial genes at contig boundaries of its largely unfinished genome sequence and which could account for a larger artificial chemosensory gene content in *C. remanei*. Second, genome assembly of *C. remanei* and *C. brenneri* is hampered by high levels of heterozygosity [49], which can also lead to inflated gene numbers due to multiple alleles. Third, and this applies to all non-*C. elegans* genomes, gene models are currently of considerably less quality for the newly sequenced species, which means that gene numbers reported here might change significantly in the near future and should be interpreted with caution. Indeed, our detailed study of the ABC transporter gene family C (Figure 5) suggests that imperfect gene models are a major problem in the newly sequenced *Caenorhabditis* species. Guided by our comparative gene family classification strategy, we identified six gene models in *C. briggsae* and *C. remanei* that are likely defective, causing artificially inflated numbers of ABC transporter genes reported by TRIBE-MCL in these species.

As more genomes are sequenced and genes annotated, more users will search for appropriate methods and strategies for genome-wide gene family classification. We showed that currently available programs for automatic sequence-based gene family classification can reconstruct manually curated gene families quite accurately. However, even the best performing programs need to be adjusted for different protein families and data sets to yield optimal performance. We demonstrated that a comparative approach is helpful in this context: by adjusting program parameters such that reference gene families of well-studies species are classified correctly, it is possible to simultaneously and correctly classify genes of the same families in other, less-studied (or newly sequenced) species. Many gene families have been worked up with great detail and large efforts in the past, providing a rich substrate for comparative gene family classification to work with. We predict this approach to be very useful in the future when many newly sequenced species will become available.

## Materials and Methods

### Search and selection of gene family classification programs

The search for gene family classification programs was mainly conducted within the body of PubMed-listed literature. Additional methods were identified by Internet search via Google, looking for terms including “protein family classification”, “gene family classification”, and “sequence clustering”.

Three programs were not considered for performance comparison albeit a stand-alone program was available. GeneRAGE [33] was excluded because of long runtimes on our system. Our attempt to cluster the *C. elegans* proteome (20,140 proteins, WS180) with GeneRAGE failed for an unknown reason after 20 days of runtime. The second program ProClust [27], [35] did not compile on our system (Linux version 2.6.23.17–88.fc7 (mockbuild@xenbuilder4.fedora.phx.redhat.com), gcc version 4.1.2 20070925 (Red Hat 4.1.2–27) due to compiler incompatibilities. ProClust source code was obtained from http://promoter.mi.uni-koeln.de/~proclust/(version 1.0.1). BAG [30] was excluded due to license requirements.

### Reference data sets (benchmarks)

Reference classification for *C. elegans* chemosensory genes was obtained from WS180 gene class annotations. Gene family names, family sizes, and references are shown in Table S1. We noticed a small increase in gene numbers for most chemosensory gene families in the WS180 release (release date September 17, 2007) in comparison to gene numbers reported by Robertson and Thomas [36], probably due to refined annotation. *C. elegans* ABC transporter gene families were derived from Zhao *et al.* [45] by mapping gene names reported by Zhao *et al*. to the WS180 data set. ABC transporter families and their sizes are shown in Table S2.

### Measurement of classification performance

To assess the performance of a given classification result, we compute sensitivity, specificity, and Jaccard index for each known gene family in our reference set as a function of its overlap with predicted gene families (Figure S1). The overlap is quantified in terms of number of genes that are found both in the known and the predicted gene family (TP = true-positives), number of genes that are found in the predicted but not in the known gene family (FP = false-positives), and the number of genes that are found in the known but not in the predicted gene family (FN = false-negative). Note that we count genes not assigned to a known family in the reference classification as false-positives. Sensitivity is computed as TP/(TP+FN) and is high if most genes of a known gene family are found within a predicted gene family. Specificity is computed as TP/(TP+FP) and is high if most genes of a predicted gene family are found within a known gene family. High Jaccard index is computed as TP/(TP+FP+FN) and is high if known and predicted families roughly contain identical genes. If a known gene family overlaps with multiple predicted gene families, sensitivity, specificity, and Jaccard index for that known gene family correspond to the overlapping family with the highest Jaccard index (“maximum overlap” rule).

To reflect classification performance across all gene families in a reference set, we computed both the unweighted and the weighted average Jaccard index. The weighted average Jaccard index is weighted by family size and gives more weight to larger gene families and less weight to smaller gene families. High *weighted* averages are only achieved if the absolute number of misclassified genes is low.

### BLAST all-vs-all comparison

*C. elegans* protein sequences were obtained from WormBase WS180 (23,511 proteins). Only longest isoforms were kept (20,140 proteins). BLASTP all-vs-all comparison was performed with the NCBI BLAST package v2.2.19 with default parameters (E-value ≤10, filter query sequence  =  on). For the *Caenorhabditis* five-species comparison, additional protein sequences of *C. briggsae* (21,978), *C. remanei* (31,518), *C. brenneri* (30,702), and *C. japonica* (25,870) were obtained from WormBase WS204 and pooled with *C. elegans* WS180 protein sequences in one FASTA file. No filtering for longest isoforms was performed for non-*C. elegans* proteins. BLASTP all-vs-all comparison was performed on the combined FASTA file (130,208 proteins; same parameters as for *C. elegans* comparison).

### TRIBE-MCL

MCL version 08-312 was obtained from http://www.micans.org/mcl/. Results in Figure 1 and 2 were generated by the following procedure: *C. elegans* all-vs-all BLAST hits with E-value ≤ 1e-10 were inputted to *mcxload* as suggested by the MCL manual (*-abc - —stream-neg-log -stream-tf ‘mul(0.4343), ceil(200)’ —stream-mirrorlist*). The resulting. mci file was clustered with *mcl* at varying inflation values, ranging from 1.1 to 5.0 (step size 0.1). Maximum number of iterations (*-L*) was set to 500 to prevent overly long runtimes for some inflation values. The number of processors (*-te*) was set to 4 to speed up computation. All other *mcl* parameters were left default. The average runtime of *mcl* on the *C. elegans*-only data set was 34 seconds.

The same procedure was applied for clustering the larger, five-species data set (Figure 4; Table 2 and 3), but here the E-value threshold used for filtering the BLAST output prior to clustering was allowed to varybetween 1e-50 and 0.1. We ran TRIBE-MCL with all possible combinations of E-value and inflation value (32 E-values ×39 inflation values  = 1,248 runs). The combined five-species data set comprised 130,208 proteins in total, including proteins from *C. elegans* (WS180, 20,140 proteins), *C. briggsae* (WS204, 21,978 proteins), *C.remanei* (WS204, 31,518 proteins), *C. brenneri* (WS204, 30,702 proteins), and *C. japonica* (WS204, 25,870 proteins). The inclusion of *C. elegans* WS180 instead of the latest WS204 allowed us to assess classification performance against our *C. elegans* benchmark data set.

### MC-UPGMA

MC-UPGMA version 1.0.0 was downloaded from http://www.protonet.cs.huji.ac.il/mcupgma/. Results in Figure 1 and 2 were generated by the following procedure: *C. elegans* BLAST all-vs-all hits were filtered for hits with E-value ≤ 1e-10. Reciprocal hits were symmetrified by considering only the one with the lower E-value (better hit). Sparse values in the similarity matrix (i.e. proteins that had no similarity with E-value ≤ 1e-10) were assigned a similarity value of 10.0 (-*max-distance* parameter of the program). After clustering, the produced hierarchical tree was cut at varying but uniform similarity thresholds ( = E-value tree-cutoff), ranging from 1e-50 to 9.9. All proteins found in sub-trees below that similarity threshold were assigned to the same final cluster. The same procedure was applied for clustering the larger five-species data set, but, as in the case of TRIBE-MCL, the E-value threshold for filtering the five-species BLAST output was now allowed to vary between 1e-50 and 0.1. All combinations of BLAST E-values and E-value tree-cutoffs were tested. Average runtime for clustering the *C. elegans* proteome with MC-UPGMA was 6 seconds.

### gSPC

The gSPC program version 1.15 was obtained from the authors upon request. As before, *C. elegans* BLAST all-vs-all hits were filtered for hits with E-value ≤ 1e-10. Reciprocal hits were symmetrified by considering only the one with the lower E-value (better hit). gSPC requires distances instead of similarities for clustering, which we computed as 200-(min(200,-log_10_(E-value))). The distance between identical proteins and between proteins with an E-value of 0 was defined as 0. The *kNN* parameter was varied between 10 and 300 with step size 10. The *temperature* parameter ranged between 1e-05 (minimum) and 0.1 (maximum) with step size 0.005. Other clustering parameters were kept constant (*iterations* = 2000; *spins* = 20;*parallel* = 4;*joint* = 1;*gamma* = 0.5;*symmetric* = 1). The average runtime of gSPC per parameter setting was 12 s. Best result on chemosensory genes (Figure 1) was achieved at *temperature* = 0.02001 and *kNN* = 40. Best result on ABC transporters (Figure 2) was achieved at *temperature* = 0.05001 and *kNN* = 20.

### FORCE

A stand-alone JAVA implementation of FORCE was obtained from http://gi.cebitec.uni-bielefeld.de/comet/force/(v1.0 beta5). FORCE incorporates a genetic algorithm that finds optimal values for parameters automatically. No parameters were thus set by us except the *–cutoff* parameter, which was set to m3.4 as suggested in the manual. Input data were again BLAST pair-wise protein sequence similarities, which were generated as described previously (E-value ≤ 1e-10). Self-similarities of proteins were excluded. FORCE required 3.5 GB of RAM assigned to the JAVA virtual machine to be run successfully. A more time and space efficient cost matrix calculator is available at http://gi.cebitec.uni-bielefeld.de/comet/force/, which we did not use in this analysis. Time required for clustering the *C. elegans* proteome was 25 hours. We tested all three different cost models and obtained identical results.

### HomoClust

Linux executables version 1.1 were downloaded from http://mars.csie.ntu.edu.tw/~cychen/HC/HomoClust.htm. The input file for HomoClust was generated from the same *C. elegans* BLAST all-vs-all comparison as used previously. Only hits with E-value ≤ 1e-10 were considered. Self-similarities were ignored. Similarity values corresponded directly to E-values as determined by BLAST. No symmetrification of similarity values was performed as this was not required by HomoClust. The two key parameters of HomoClust are Sim_down-th_ and Sim_up-th_, which specify the minimum and maximum sequence similarity, respectively, used for evaluating the homogeneity of clusters in the first phase of the algorithm. For both parameters, we tested values ranging from 0 to 250 with step size 10. Other fixed parameters were ‘*–s evalue’* and ‘*–a HomoClust’*. All other parameters were left default. Reported homogeneous clusters were interpreted as putative gene families. Cutting the reported cluster hierarchy at other, varying similarity thresholds (as was done for MC-UPGMA) was not possible in case of HomoClust, because no cluster similarity or distance values were provided in the program output. The average runtime per parameter set tested was 9 seconds.

### CLUSS

CLUSS version 3.0 was downloaded from http://prospectus.usherbrooke.ca/CLUSS/Download/SRC/CLUSS_3.0/CLUSS.rar. CLUSS 3.0 allows selecting older program version (1.0 and 2.0) at startup and we tested all three of them (the *Kmer* program version was not tested). CLUSS was run directly with *C. elegans* WS180 protein sequences as input without prior BLAST comparison (no external similarity measure required by the program). Other parameters were: *substitution matrix*  =  BLOSOM62; *redundant sequences*  =  withdraw; *phylogenetic tree*  =  one tree for each subfamily. CLUSS 2.0 gave slightly better results than CLUSS 1.0 and thus CLUSS 2.0 results were used. CLUSS 3.0 crashed twice after one week runtime with the error ‘too many iterations in eigenvectors searches’. Execution time was 12 and 55 hours for CLUSS 1.0 and CLUSS 2.0, respectively. Note that this time includes the generation of pairwise sequence similarities, because CLUSS was not run with precomputed BLAST results.

### BLASTClust

BLASTClust is part of the NCBI BLAST package and was downloaded from http://www.ncbi.nlm.nih.gov/BLAST/download.shtml (version 2.2.19). Minimum sequence similarity threshold was specified in terms of percent identity (*-S* parameter) and varied between 10 and 80 with step size 10. Minimum alignment length coverage (*-L* parameter) varied between 0.1 and 0.9 with step size 0.1. E-value threshold in the BLASTClust config file (*-e* parameter) was set to 1e-10. Other fixed command line parameters were *–p T* (input is protein sequence) and *–a 5* (number of CPUs). All other parameters default. We generated a hit-list file (containing neighboring proteins above threshold) at first run of BLASTClust (*-s* parameter) and used this file for subsequent runs to speed up computation (*-r* parameter). The first run of BLASTClust took 30 minutes (including the generation of the hit-list file). Subsequent runs of BLASTClust finished in fewer than one second.

### Phylogenetic analysis

The phylogenetic tree in Figure 5C was produced with MEGA4 [50]. We used ClustalW [51] to construct a multiple alignment (default parameters) of both known and putative new ABC transporter family C genes. For the identified split gene models (Figure 5A and 5B) we included the protein sequence encoded by the corrected, longer gene models. Columns containing gaps as well as immediately adjacent columns were removed from the alignment before tree construction. The phylogenetic tree was produced by the minimum evolution method and 1000 bootstrap iteration.

## Supporting Information

Figure S1 Classification performance measures as a function of overlap between known and predicted gene families. False-positives, true-positives, and false-negatives refer to number of genes. Genes not assigned to a family in the reference classification are counted as false-positives.
(0.18 MB TIF)

Figure S2 Heat-map revealing low sequence similarities between srv family members. The lower-left half of the matrix shows pair-wise sequence similarities determined by BLAST (E-value threshold 10). The upper-right half of the matrix shows pair-wise sequence similarity determined by PSI-BLAST. Only PSI-BLAST finds sequence similarity among all proteins within that family. Numbers within squares correspond to -log10(E-value). Dark red indicates high sequence similarity, light red indicates low sequence similarity. White (empty) squares indicate that no sequence similarity has been reported.
(0.97 MB TIF)

Figure S3 Heat-map showing reduced but existing sequence similarity between str and srj family members. Figure produced with MultiExperiment Viewer [52].
(0.95 MB TIF)

Text S1 Brief review and results summary of the seven selected programs for performance comparison
(0.04 MB DOC)

Table S1 C. elegans chemosensory gene families used as reference classification for performance evaluation.
(0.13 MB DOC)

Table S2 C. elegans ABC transporter gene families used as reference classification for performance evaluation.
(0.03 MB DOC)

Table S3 Five-species TRIBE-MCL classification result of Caenorhabditis chemosensory genes. This table contains all genes found in chemosensory gene clusters (defined as TRIBE-MCL clusters that contain at least one annotated C. elegans chemosensory gene).
(0.74 MB XLS)

Table S4 Five-species TRIBE-MCL classification result of Caenorhabditis ABC transporter genes. This table contains all genes found in ABC transporter gene clusters (defined as TRIBE-MCL clusters that contain at least one annotated C. elegans ABC transporter gene).
(0.06 MB XLS)
