# Supplementary figures and images for: Genome-Wide Comparative Gene Family Classification
Source: PLoS One. 2010 Oct 15;5(10):e13409. doi: 10.1371/journal.pone.0013409 (PMC2955529; doi:10.1371/journal.pone.0013409)

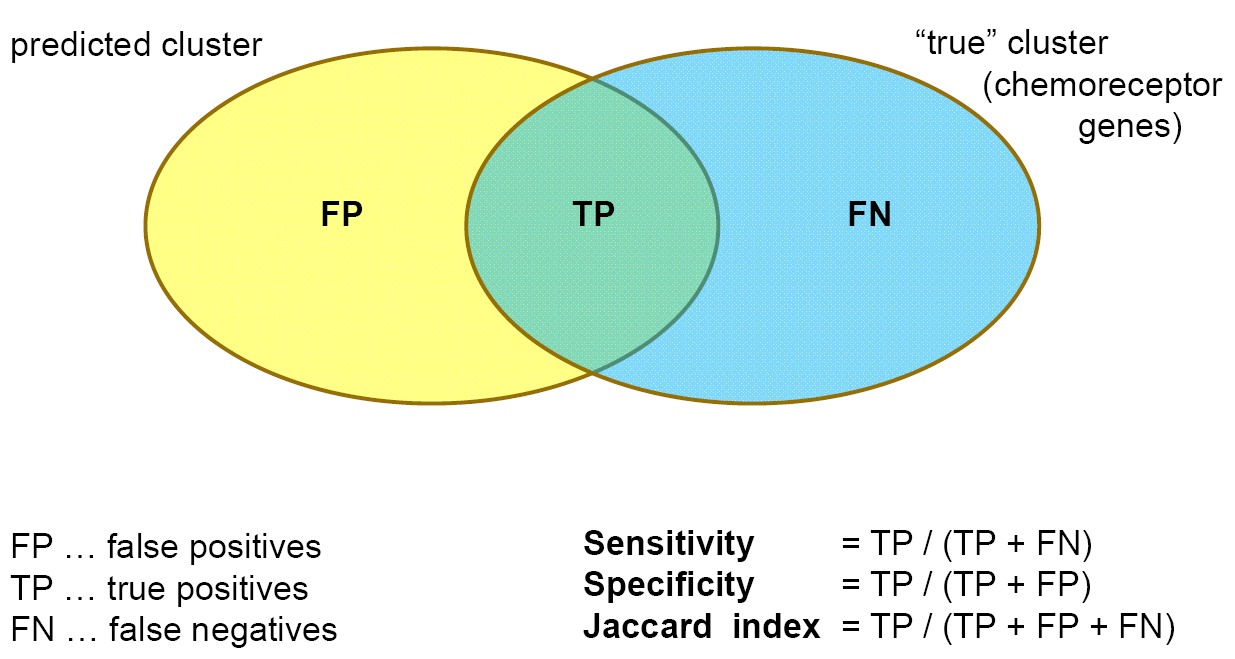

Supplement: Figure S1 — Classification performance measures as a function of overlap between known and predicted gene families. False-positives, true-positives, and false-negatives refer to number of genes. Genes not assigned to a family in the reference classification are counted as false-positives. (0.18 MB TIF) [file pone.0013409.s001.tif]

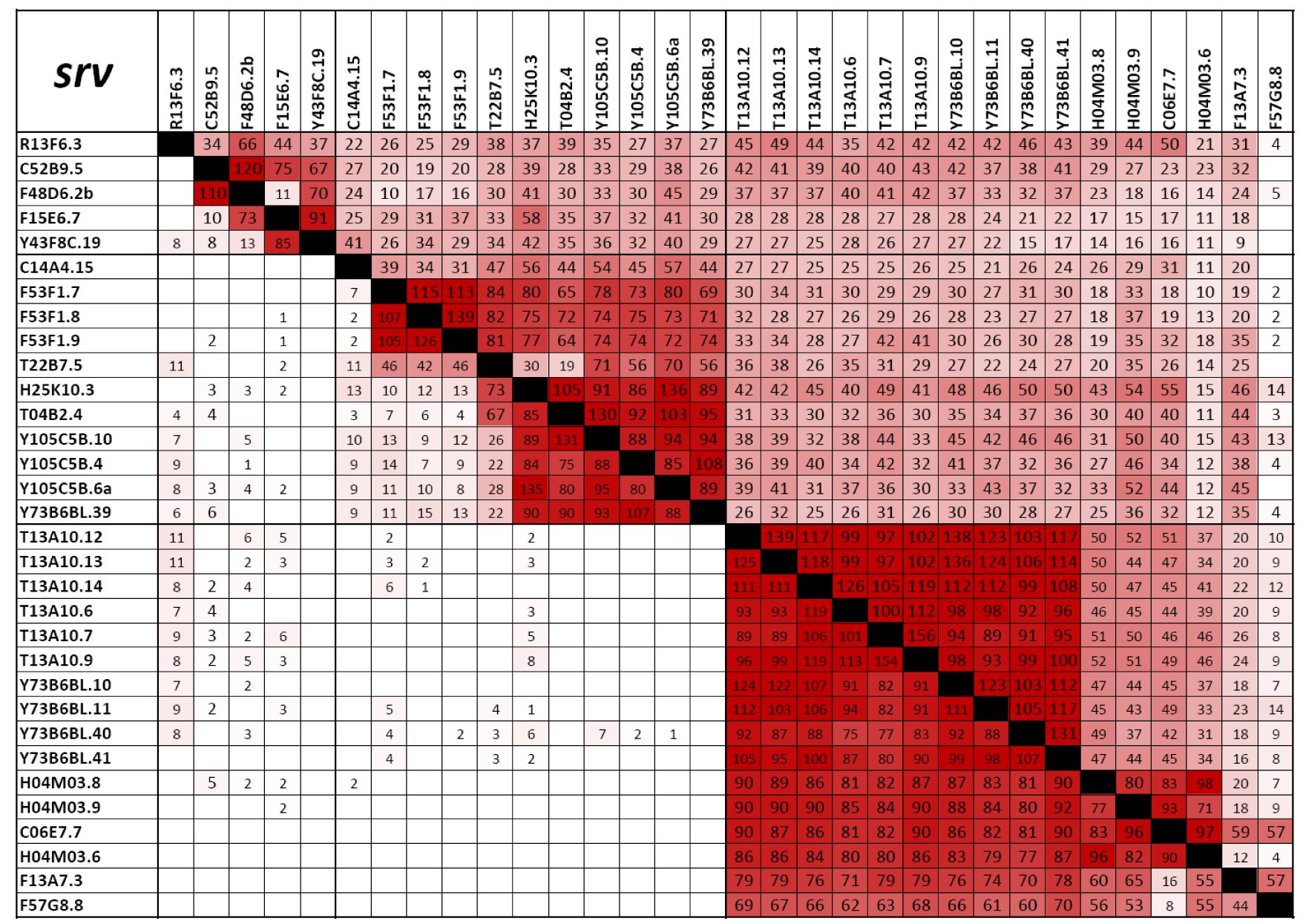

Supplement: Figure S2 — Heat-map revealing low sequence similarities between srv family members. The lower-left half of the matrix shows pair-wise sequence similarities determined by BLAST (E-value threshold 10). The upper-right half of the matrix shows pair-wise sequence similarity determined by PSI-BLAST. Only PSI-BLAST finds sequence similarity among all proteins within that family. Numbers within squares correspond to -log10(E-value). Dark red indicates high sequence similarity, light red indicates low sequence similarity. White (empty) squares indicate that no sequence similarity has been reported. (0.97 MB TIF) [file pone.0013409.s002.tif]

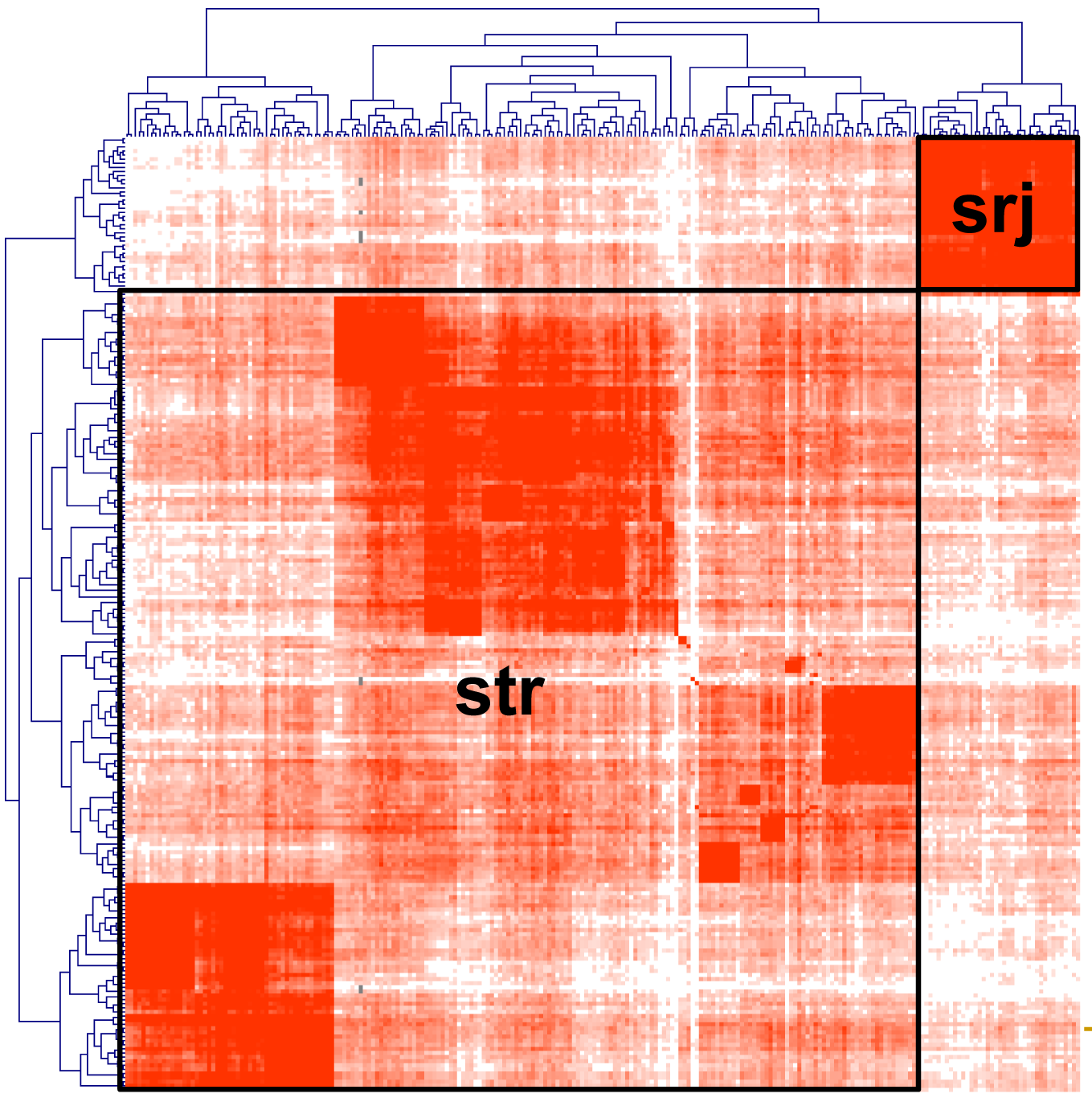

Supplement: Figure S3 — Heat-map showing reduced but existing sequence similarity between str and srj family members. Figure produced with MultiExperiment Viewer [52]. (0.95 MB TIF) [file pone.0013409.s003.tif]
