# Supplementary material for: Genome-Wide Comparative Gene Family Classification
Source: PLoS One. 2010 Oct 15;5(10):e13409. doi: 10.1371/journal.pone.0013409 (PMC2955529; doi:10.1371/journal.pone.0013409)
Supplement: Text S1 — Brief review and results summary of the seven selected programs for performance comparison (0.04 MB DOC) [file pone.0013409.s004.doc]

# Text S1: Supplemental Information

**Brief review and results summary of the seven selected programs for performance comparison**

**TRIBE-MCL** [1], a method that uses an efficient Markov cluster algorithm for clustering and that repeatedly ranked among top-performing methods in previous comparisons, reproduced 17 out of 22 chemosensory gene families and 6 out of 8 ABC transporter gene families with good quality (Jaccard index > 0.8) and was among the top-three methods on both our data sets. Interestingly, **MC-UPGMA** [2], a recently introduced memory-constraint implementation of the popular UPGMA clustering algorithm (a hierarchical buttom-up average-linkage clustering algorithm) that was shown to outperform simpler single-linkage algorithms on very large data sets [2], was the best performing method in our comparison and even slightly outperformed TRIBE-MCL. Global super-paramagnetic clustering (**gSPC**) [3], previously shown to outperform TRIBE-MCL up to 30% on the author’s own data sets, matched the overall performance of MC-UPGMA and TRIBE-MCL on the chemosensory gene data set, but achieved lower performance on ABC transporters. **FORCE** [4], a graph-based method that applies the concept of weighted cluster editing and that was previously shown to outperform spectral clustering, TRIBE-MCL, GeneRAGE, hierarchical clustering, and affinity propagation on the ASTRAL data set [4], reproduced the majority of chemosensory gene families with good quality and in agreement with MC-UPGMA and TRIBE-MCL, but underperformed on four families (*srd*, *sre*, *srsx*, and *srx*) due to reduced sensitivity. On ABC transporter genes, FORCE underperformed due to low specificity, suggesting that the clustering parameters automatically determined by FORCE for clustering the *C. elegans* proteome were suitable for the divergent chemosensory genes but not for the more closely related ABC transporters. **HomoClust** [5] is a two-phase single-linkage hierarchical clustering algorithm that was shown to outperform both traditional single-linkage and average-linkage algorithms for clusters associated with large families [5]. **BLASTClust** [6] is part of the NCBI BLAST package and clusters BLAST results at user-defined similarity thresholds by single-linkage clustering. Both HomoClust and BLASTClust grouped ABC transporter genes with high accuracy, but showed moderate to poor performance on the majority of chemosensory gene families (Jaccard index <= 0.75) due to reduced sensitivity. **CLUSS** [7], the only alignment-independent algorithm in the set, was previously shown to outperform BLASTClust, TRIBE-MCL, and gSPC on author-selected gene families, but performed poor on both of our data sets. CLUSS does not seem to be an appropriate method for clustering complete proteomes, as it partitions the *C. elegans* proteome into only few and large clusters (648 clusters with average size of 31.1 genes) that are in bad agreement with both our reference sets. The time required to classify the complete *C. elegans* proteome varied significantly between programs, ranging from < 1 s (average value per tested parameter setting) for the single-linkage clustering algorithm BLASTClust to 55h for CLUSS.

**Supplementary analysis of poorly reconstructed gene families**

Only few gene families were reconstructed poorly by both MC-UPGMA and TRIBE-MCL, such as the chemosensory gene families *srv* (Jaccard index 0.50), *srg* (0.47), and *srj* (0.15) (Figure 1A). For the *srv* and *srg* gene families we observe perfect specificity (1.00) but low sensitivity (*srv*: 0.50; *srg*: 0.47), which suggests that members of these families have divergent sequences. Indeed, BLAST fails to detect sequence similarity between many members of the *srv* gene family at E-value threshold 1e-10 (Figure S2, below diagonal). A more sensitive sequence-based search with PSI-BLAST revealed significant sequence similarity between all *srv* family members (Figure S2, above diagonal), underlining the importance of remote homology detection in gene family classification. A similar situation is observed in gene family *srg* (data not shown). A different problem accounted for misclassification of the *srj* gene family (sensitivity 1.00; specificity 0.15). We found that in this case the whole gene family groups together with the larger *str* gene family, which explains also the reduced specificity for the *str* family (0.73). The heat map shown in Figure S3 suggests that there is enough structure in the data to correctly resolve gene families *srj* and *str*, and indeed we observed a correct separation between *srj* and *str* at TRIBE-MCL inflation values > 3.0 (data not shown).

# References

1. Enright AJ, Dongen SV, Ouzounis CA (2002) An efficient algorithm for large-scale detection of protein families. Nucleic Acids Res 30: 1575--1584.

2. Loewenstein Y, Portugaly E, Fromer M, Linial M (2008) Efficient algorithms for accurate hierarchical clustering of huge datasets: tackling the entire protein space. Bioinformatics 24: i41--i49.

3. Tetko IV, Facius A, Ruepp A, Mewes H-W (2005) Super paramagnetic clustering of protein sequences. BMC Bioinformatics 6: 82.

4. Wittkop T, Baumbach J, Lobo FP, Rahmann S (2007) Large scale clustering of protein sequences with FORCE -A layout based heuristic for weighted cluster editing. BMC Bioinformatics 8: 396.

5. Chen C-Y. Detecting homogeneity in protein sequence clusters for automatic functional annotation and noise detection; 2005. pp. 2 pp.--.

6. BLASTCLUST - BLAST score-based single-linkage clustering.

7. Kelil A, Wang S, Brzezinski R, Fleury A (2007) CLUSS: clustering of protein sequences based on a new similarity measure. BMC Bioinformatics 8: 286.

8. Saeed AI, Sharov V, White J, Li J, Liang W, et al. (2003) TM4: a free, open-source system for microarray data management and analysis. Biotechniques 34: 374-378.
