# Supplementary material for: Genome-Wide Comparative Gene Family Classification
Source: PLoS One. 2010 Oct 15;5(10):e13409. doi: 10.1371/journal.pone.0013409 (PMC2955529; doi:10.1371/journal.pone.0013409)
Supplement: Table S1 — C. elegans chemosensory gene families used as reference classification for performance evaluation. (0.13 MB DOC) [file pone.0013409.s005.doc]

**Table S1:** *C. elegans* chemosensory gene families used as reference classification for performance evaluation.

| **Chemosensory gene family** | **Size** | **Reference** |
| --- | --- | --- |
| sra | 34 | [1,2,3] |
| srab | 23 | [2,4] |
| srb | 16 | [2,3,5] |
| srbc | 73 | [2,5] |
| srd | 66 | [2,3,5] |
| sre | 53 | [2,3,5] |
| srg | 62 | [2,3,5] |
| srh | 223 | [2,6] |
| sri | 60 | [2,7,8] |
| srj | 39 | [2,7,8] |
| srm | 6 | [5] |
| srn | 1 | [5] |
| srr | 10 | [5] |
| srsx | 37 | [2,5] |
| srt | 66 | [2,9] |
| sru | 40 | [2,5] |
| srv | 32 | [2,5] |
| srw | 119 | [2,5] |
| srx | 106 | [2,5] |
| srxa | 17 | [2,5] |
| srz | 67 | [2,10] |
| str | 193 | [2,7,8] |

The classification is based on gene class annotations taken from Wormbase (WS180). References refer to publications in which the gene family has been first described.

# References

1. Stein LD, Bao Z, Blasiar D, Blumenthal T, Brent MR, et al. (2003) The genome sequence of Caenorhabditis briggsae: a platform for comparative genomics. PLoS Biol 1: E45.

2. Thomas JH, Robertson HM (2008) The Caenorhabditis chemoreceptor gene families. BMC Biol 6: 42.

3. Troemel ER, Chou JH, Dwyer ND, Colbert HA, Bargmann CI (1995) Divergent seven transmembrane receptors are candidate chemosensory receptors in C. elegans. Cell 83: 207-218.

4. Chen N, Pai S, Zhao Z, Mah A, Newbury R, et al. (2005) Identification of a nematode chemosensory gene family. Proc Natl Acad Sci U S A 102: 146-151.

5. Robertson HM, Thomas JH (2006) The putative chemoreceptor families of C. elegans. WormBook: 1--12.

6. Robertson HM (2000) The large srh family of chemoreceptor genes in Caenorhabditis nematodes reveals processes of genome evolution involving large duplications and deletions and intron gains and losses. Genome Res 10: 192-203.

7. Robertson HM (1998) Two large families of chemoreceptor genes in the nematodes Caenorhabditis elegans and Caenorhabditis briggsae reveal extensive gene duplication, diversification, movement, and intron loss. Genome Res 8: 449-463.

8. Robertson HM (2001) Updating the str and srj (stl) families of chemoreceptors in Caenorhabditis nematodes reveals frequent gene movement within and between chromosomes. Chem Senses 26: 151-159.

9. Thomas JH (2006) Analysis of homologous gene clusters in Caenorhabditis elegans reveals striking regional cluster domains. Genetics 172: 127-143.

10. Thomas JH, Kelley JL, Robertson HM, Ly K, Swanson WJ (2005) Adaptive evolution in the SRZ chemoreceptor families of Caenorhabditis elegans and Caenorhabditis briggsae. Proc Natl Acad Sci U S A 102: 4476-4481.
