# Supplementary material for: Genome-Wide Comparative Gene Family Classification
Source: PLoS One. 2010 Oct 15;5(10):e13409. doi: 10.1371/journal.pone.0013409 (PMC2955529; doi:10.1371/journal.pone.0013409)
Supplement: Table S2 — C. elegans ABC transporter gene families used as reference classification for performance evaluation. (0.03 MB DOC) [file pone.0013409.s006.doc]

**Table S2:** *C. elegans* ABC transporter gene families used as reference classification for performance evaluation.

| **ABC transporter gene family** | **Size** |
| --- | --- |
| A | 7 |
| B | 24 |
| C | 9 |
| D | 5 |
| E | 1 |
| F | 3 |
| G | 9 |
| H | 2 |

This data set was derived from Zhao *et al.* [1] by mapping the gene names reported by Zhao *et al*. to the WS180 data set.

# References

1. Zhao Z, Thomas JH, Chen N, Sheps JA, Baillie DL (2007) Comparative genomics and adaptive selection of the ATP-binding-cassette gene family in caenorhabditis species. Genetics 175: 1407-1418.
